# Circulating resistin levels and obesity-related cancer risk: A meta-analysis

**DOI:** 10.18632/oncotarget.11034

**Published:** 2016-08-04

**Authors:** Wei-Jing Gong, Wei Zheng, Ling Xiao, Li-Ming Tan, Jian Song, Xiang-Ping Li, Di Xiao, Jia-Jia Cui, Xi Li, Hong-Hao Zhou, Ji-Ye Yin, Zhao-Qian Liu

**Affiliations:** ^1^ Department of Clinical Pharmacology, Xiangya Hospital, Central South University, Changsha, P. R. China; ^2^ Institute of Clinical Pharmacology, Central South University, Hunan Key Laboratory of Pharmacogenetics, Changsha, P. R. China; ^3^ Hunan Province Cooperation Innovation Center for Molecular Target New Drug Study, Hengyang, P. R. China; ^4^ Department of Otolaryngology, Xiangya Hospital, Central South University, Changsha, P.R. China; ^5^ Department of Pharmacy, Xiangya Hospital, Central South University, Changsha, P.R. China

**Keywords:** circulating resistin levels, obesity-related cancer, meta-analysis

## Abstract

Resistin levels have been reported to be abnormal in obesity-related cancer patients with epidemiological studies yielding inconsistent results. Therefore, a meta-analysis was performed to assess the association between blood resistin levels and obesity-related cancer risk. A total of 13 studies were included for pooling ORs analysis. High resistin levels were found in cancer patients (OR= 1.20, 95% CI= 1.10-1.30). After excluding one study primarily contributing to between-study heterogeneity, the association between resistin levels and cancer risk was still significant (OR=1.18, 95% CI = 1.09-1.28). Stratification analysis found resistin levels were not associated with cancer risk in prospective studies. Meta-regression analysis identified factors such as geographic area, detection assay, or study design as confounders to between-study variance. The result of 18 studies of pooling measures on SMD analysis was that high resistin levels were associated with increased cancer risk (SMD = 0.94, 95% CI = 0.63-1.25), but not in the pooling SMD analysis of prospective studies. Except for the studies identified as major contributors to heterogeneity by Galbraith plot, resistin levels were still higher in cancer patients (SMD = 0.75, 95% CI = 0.63-0.87) in retrospective studies. Meta-regression analysis found factors, such as geographic area, BMI-match, size, and quality score, could account for 66.7% between-study variance in pooling SMD analysis of retrospective studies. Publication bias was not found in pooling ORs analysis. Our findings indicated high resistin levels were associated with increased obesity-related cancer risk. However, it may not be a predictor.

## INTRODUCTION

Obesity and diabetes are considered as important risk factors of cancers. According to a population-based study in 2012, a quarter of the cancer cases possess high body-mass index (BMI) [1]. Among them, prostate, breast, colorectal, thyroid, renal, endometrial, pancreatic and esophageal cancers are identified as obesity-related cancers by a number of epidemiological studies and meta-analyses [2]. Also, individuals with diabetes have significant higher risk of cancer compared with no diabetes [3]. However, the mechanisms underlying the association between obesity or diabetes and cancer development are currently not fully elucidated.

Resistin was first identified by a screening of adipocyte products that were decreased by rosiglitazone in mice. It was considered as the potential link between obesity and diabetes [4]. Resistin expression in prostate epithelial cells was also found to be higher in patients with prostate cancer, compared with that in those with benign prostate hyperplasia [5]. Additionally, serum resistin levels were reported to be increased in several cancers, such as breast and colorectal cancers. Studies revealed resistin could promote the proliferation, angiogenesis, and metastasis of cancer cells by stimulating specific signaling pathways including p38 MAPK/NF-kB and PI3K/Akt [6–8]. Although many studies provided evidence that high resistin levels were associated with the risk of obesity-associated malignancies, some studies observed different results. Many studies showed resistin levels were similar, even lower in cancer patients compare with normal controls. The reasons underlying these heterogeneous findings need to be investigated.

To the best of our knowledge, no systematic review evaluated the association of blood resistin levels with obesity-related cancer risk. More convincing evidence is needed to reveal the role of resistin in obesity-related cancers. The present study aimed to evaluate the association of circulating resistin levels with the risk of obesity-related cancers by conducting a meta-analysis.

## RESULTS

### Literature search

**Figure 1:**
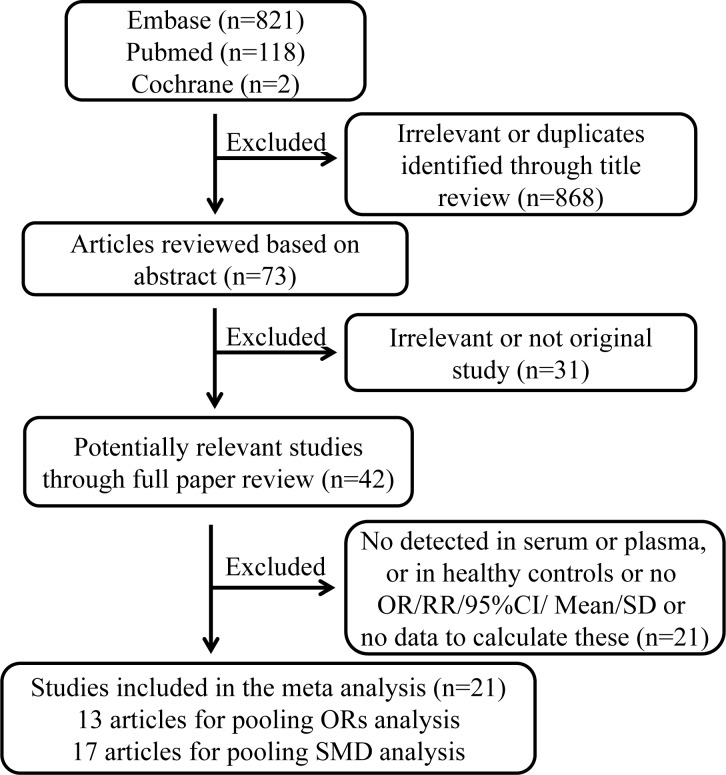
Procedure of article selection

The procedure of literature selection is presented in Figure 1. We identified 42 potentially relevant papers concerning resistin in relation to cancer risk. 9 papers were excluded because circulating resistin levels were not measured in serum or plasma of the healthy controls or obesity-related cancers. 12 papers were excluded because that they did not provide sufficient information. Finally, for pooling odds ratios (ORs) analysis, 13 articles were included involving 9 retrospective studies and 4 prospective studies [9–21]. With regard to the pooling measures on standardized mean difference (SMD), 17 papers containing 14 retrospective articles and 3 prospective articles [9–14, 19–29] were included.

### Characteristics of included studies

13 studies for meta-analysis performed on ORs were published from 2007 to 2016, involving 2756 cases and 3350 controls. 8 and 4 articles focused on breast and colorectal cancer, respectively [9–20]. 6 articles were conducted in Asia [9, 13–17], 3 in Europe [11, 12, 21], 3 in the USA [18–20], and 1 in Africa [10]. The ORs of most studies were adjusted for age and BMI. Circulating resistin levels were measured by enzyme-linked immunosorbent assay (ELISA) in 10 studies [9–17, 21], and by Human Adipokine Panel in 3 studies [18–20]. The quality score of studies ranged from 5 stars to 8 stars according to the 9-star Newcastal-Ottawa Scale [30]. General characteristics of the involved studies are shown in Table 1.

**Table 1:** Characteristics of studies included in pooling ORs analysis

Author	Year	Country	Cancer Type	Control Source	Study Design	Detection Assay	NOS Score	Case/Control	Adjusted OR (95% CI)	Adjustments
Alokail	2013	Saudi Arabia	BC	HB	Retrospective case-control	ELISA	6	56/53	1.90 (0.62-5.70)	age, menopausal status of menarche
Aly	2013	Egypt	BC	HB	Retrospective case-control	ELISA	5	35/40	1.26(1.21-1.93)	No
Dalamaga	2013	Greece	BC	HB	Retrospective case-control	ELISA	6	102/102	1.17(1.03-1.34)	age, date of diagnosis, education, BMI, waist circumference, family history of cancer, use of exogenous hormones, smoking history, adiponectin and leptin concentration, inflammatory markers, alcohol consumption, smoking status
Danese	2012	Italy	CC	HB	Retrospective case-control	ELISA	6	40/40	1.33(1.03-1.72)	age, sex, BMI, lifestyle parameters
Gaudet^1^	2010	United States	BC	PB	Prospective nest case-control	Human Adipokine Panel	7	234/231	1.09(0.58-2.08)	age, BMI, number of births, age at first full-term birth, age at menopause, and current postmenopausal hormone use
Gunter^1^	2015	United States	BC	PB	Prospective case-cohort	Human Adipokine Panel	6	875/820	1.00(0.81-1.22)	age, ethnicity, alcohol consumption, family history of breast cancer, parity, year of menstrual cycling, age at first child's birth, use of hormone therapy, endogenous estradiol levels, history of benign breast disease, BMI and physical activity
Ho^1^	2012	United States	CC	PB	Prospective case-cohort	Human Adipokine Panel	6	427/797	0.89(0.58-1.38)	age, race, smoking status, ever had colonoscopy, estrogen level, insulin, waist circumference
Hou	2007	China	BC	HB	Retrospective case-control	ELISA	6	80/50	1.34(1.11-2.35)	NA
Kang	2007	Korea	BC	HB	Retrospective case-control	ELISA	6	41/43	2.77(1.40-5.50)	age, BMI, status of menopause, serum glucose and adiponectin
Liao^1^	2012	Finland	RCC	PB	Prospective nest case-control	ELISA	8	273/273	1.15(0.80-1.51)	number of years smoking, presence of hypertension, history of diabetes and physical activity
Nakajima	2010	Japan	CC	HB	Retrospective case-control	ELISA	7	115/115	2.07(1.05-4.06)	NA
Otake	2010	Japan	CC	HB	Retrospective case-control	ELISA	5	98/26	0.88(0.16-1.60)	No
Sun^1^	2010	Taiwan	BC	HB	Retrospective case-control	ELISA	7	380/760	1.77(0.90-2.64)	age, waist circumference, hormone replacement therapy use, family history of breast cancer, age at enrollment, age at menarche, age at first full-term pregnancy, parity number

1 Risk estimates were recalculated by the method proposed by Harmling et al. Abbreviations: HB= Hospital Based; PB = Population Based; OR = Odds Ratio; CI = Confidence Interval; ELISA = Enzyme-linked Immunosorbent Assay; BMI = Body Mass Index; NA = Unknown; NOS = Newcastle-Ottawa Scale; BC = Breast Cancer; CC = Colorectal Cancer; RCC = Renal Cell Cancer.

For pooling SMD analysis, 17 articles constituted 2421 cases and 2731 controls. Because 1 article consisted of 2 studies [24], a total of 18 studies were included. 8 studies were conducted in Asia [9, 13, 14, 22, 23, 26, 27, 29], 7 in Europe [11, 12, 21, 24, 25, 28], and 2 in the USA [19, 20]. 9 and 7 studies focused on breast and colorectal cancers, respectively [9–14, 19, 20, 22–24, 26–29] (Table 2).

**Table 2:** Characteristics of studies included in pooling SMD analysis

Author	Year	Country	Cancer Type	Study Design	Detection Assay	NOS Score	Cases			Controls		
							Number	Mean	SD	Number	Mean	SD
Al-Haritby	2010	Saudi Arabia	CC	Retrospective case-control	ELISA	4	60	19.44	8.46	60	5.45	2.73
Alokail	2013	Saudi Arabia	BC	Retrospective case-control	ELISA	6	56	18.9	1.2	53	15.2	1
Aly	2013	Egypt	BC	Retrospective case-control	ELISA	6	35	4.42	4.74	40	1.84	2.35
Assiri	2015	Saudi Arabia	BC	Retrospective case-control	ELISA	6	82	26.24	1.59	68	22.69	2.58
Crusistomo(a)^1^	2016	Portugal	BC	Retrospective case-control	ELISA	7	30	11.6	7.31	29	7.51	3.6
Crusistomo(b)^1^	2016	Portugal	BC	Retrospective case-control	ELISA	7	47	16.1	10.37	48	10.4	9.75
Dalamaga	2013	Greece	BC	Retrospective case-control	ELISA	6	102	11.2	6.4	102	7.7	4.85
Danese^1^	2012	Italy	CC	Retrospective case-control	ELISA	6	40	8.96	3.42	40	4.97	1.07
Diakowska	2014	Poland	EC	Retrospective case-control	ELISA	6	41	8.99	3.21	60	7.5	2.7
Gonullu^1^	2009	Turkey	CC	Retrospective case-control	ELISA	5	36	6.1	3.3	37	4.5	1.5
Gunter^1^	2015	United States	BC	Prospective case-cohort	Milliplex Human Adipokine Panel	5	875	12.1	4	821	12.3	4.3
Ho^1^	2012	United States	CC	Prospective case-cohort	Milliplex Human Adipokine Panel	6	457	12.8	4.81	834	12.3	4.3
Hou	2007	China	BC	Retrospective case-control	ELISA	6	80	26.35	5.36	50	23.32	4.75
Joshi	2014	Korea	CC	Retrospective case-control	ELISA	6	100	4.9	2.3	100	2.8	1.7
Kang	2007	Korea	BC	Retrospective case-control	ELISA	6	41	5.23	6.9	43	1.46	2
Kumor	2008	Poland	CC	Retrospective case-control	ELISA	4	36	6.79	2.41	25	3.6	1.08
Liao^1^	2012	Finland	RCC	Prospective nest case-control	ELISA	8	273	9.27	2.73	273	9.28	2.83
Tulubas	2014	Turkey	CC	Retrospective case-control	ELISA	6	30	18.77	5.09	30	13.36	6.36

1 Data was recalculated by the method proposed by Hozo et al. Abbreviations: SD = Standard Deviation; CI = Confidence Interval; ELISA = Enzyme-linked Immunosorbent Assay; NOS = Newcastle-Ottawa Scale; CI = Confidence Interval; BC = Breast Cancer; CC = Colorectal Cancer; EC = Esophageal Cancer

### Pooling of studies and subgroup analysis

**Figure 2:**
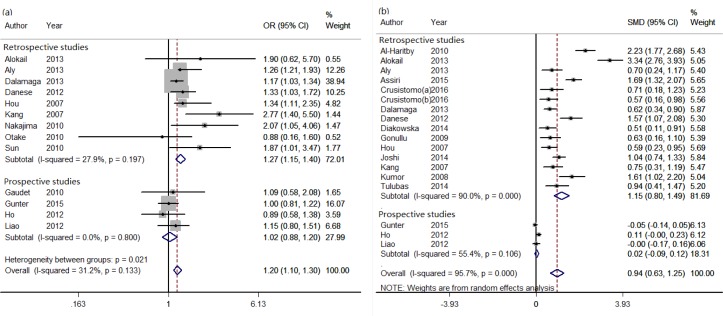
The effect of circulating resistin levels on obesity-related cancer risk in pooling ORs (a) and SMD (b) analysis

The multivariate adjusted ORs for each study and the combined OR are present in Figure 2a. The combined OR for cancer risk was 1.20 (95% CI = 1.10-1.30). There was no significant heterogeneity across the studies (*I*^2^ = 31.2%, *P* = 0.133). So a fixed-effects model was adopted (Figure 2a). Further, subgroup analysis by sample size, cancer type, geographic area, detection assay, study design, study quality, and BMI-match was conducted. High resistin levels were found to be associated with increased cancer risk in the studies of breast cancer (OR = 1.19, 95% CI = 1.08-1.31), colorectal cancer (OR = 1.25, 95% CI = 1.02-1.53), Asia (OR = 1.66, 95% CI = 1.29-2.13), Europe (OR = 1.20, 95% CI = 1.07-1.33), ELISA (OR = 1.26, 95% CI = 1.15-1.38), retrospective studies (OR = 1.27, 95% CI = 1.15-1.40). However, circulating resistin levels were similar between cases and controls in the studies of Human Adipokine Panel (OR = 0.99, 95% CI = 0.83-1.18), the USA (OR = 0.99, 95% CI = 0.83-1.18), and prospective studies (OR = 1.02, 95% CI = 0.88-1.20) (Table 3).

**Table 3:** Subgroup analysis of pooling ORs of circulating resistin and cancer risk

Subgroup	No.	Fixed Effects OR(95%CI)	*I*^2^ (%)	*P* Value^a^	*P* Value^b^
Total	13	1.20(1.10,1.30)	31.2	0.133	
Sample Size					0.122
<200	6	1.35(1.16,1.57)	8.2	0.364	
≥200	7	1.14(1.03,1.25)	29.0	0.207	
Cancer Type					0.829
Breast Cancer	8	1.19(1.08,1.31)	42.3	0.096	
Colorectal Cancer	4	1.25(1.02,1.53)	40.9	0.166	
Others	1	1.15(0.80,1,51)			
Geographic Area					0.019
Asia	6	1.66(1.29,2.13)	4.6	0.387	
Europe	3	1.20(1.07,1.33)	0.0	0.662	
USA	3	0.99(0.83,1.18)	0.0	0.849	
Africa	1	1.26(1.21,1.93)			
Detection Assay					0.041
ELISA	10	1.26(1.15,1.38)	21.3	0.247	
Human Adipokine Panel	3	0.99(0.83,1.18)	0.0	0.849	
Study Design					0.044
Retrospective Study	9	1.27(1.15,1.40)	27.9	0.197	
Prospective Study	4	1.02(0.88,1.20)	0.0	0.800	
Study quality					0.490
NOS score(7-9)	4	1.32(1.04,1.69)	24.9	0.262	
NOS score(5-6)	9	1.18(1.08,1.29)	36.9	0.123	
BMI match					0.340
Yes	9	1.23(1.09,1.39)	46.0	0.063	
No	4	1.17(1.04,1.30)	0.0	0.534	

a *P*-Value for heterogeneity within each subgroup.

b *P*-Value for heterogeneity between subgroups with meta-regression analysis

Abbreviations: No. = Number of studies; ELISA = Enzyme-linked Immunosorbent Assay; NOS = Newcastle-Ottawa Scale; OR = Odds Ratio; CI = Confidence Interval; BMI = Body Mass Index

18 studies were available to evaluate the SMD of circulating resistin levels with obesity-related cancer risk. Because of high heterogeneity (*I^2^* = 95.7%, *P* = 0.000), a random-effects model was used. Higher resistin levels were present in cancer patients (SMD = 0.94, 95% CI = 0.63-1.25) (Figure 2b). Stratification analysis found that there was no significant association between circulating resistin levels and obesity-related cancer risk in prospective studies (SMD = 0.02, 95% CI = −0.09-0.12) (Figure 2b). However, for retrospective studies, stratification analysis showed that resistin levels were always higher in cancer patients (Table 4).

**Table 4:** Subgroup analysis of pooling SMD of circulating resistin levels and obesity-related cancer risk in retrospective studies

Subgroup	Number of studies	Random-Effects SMD(95% CI)	Heterogeneity	
			I^2^ (%)	*P*
Total	15	1.15(0.80,149)	90.0	0.000
Sample Size				
<100	8	0.91(0.64,1.19)	61.0	0.012
≥100	7	1.41(0.79,2.02)	94.9	0.000
Cancer Type				
Breast Cancer	8	1.10(0.57,1.64)	92.6	0.000
Colorectal Cancer	6	1.33(0.87,1.79)	80.1	0.000
Others	1	0.51(0.11,0.91)		
Geographic Area				
Asia	8	1.39(0.82,1.95)	93.0	0.000
Europe	6	0.89(0.53,1.26)	76.6	0.001
Africa	1	0.70(0.24,1.17)		
Study Quality				
NOS Score(7-9)	3	1.00(0.24,1.75)	89.0	0.000
NOS Score(5-6)	10	1.04(0.64,1.45)	89.7	0.000
NOS Score(0-4)	2	1.95(1.35,2.55)	61.8	0.106
Resistin Levels in Controls				
0-5 ng/ml	6	1.03(0.72,1.34)	65.3	0.013
5-10ng/ml	4	1.01(0.27,1.75)	92.6	0.000
10-15ng/ml	2	0.71(0.36,1.07)	15.0	0.278
15- ng/ml	3	1.86(0.47,3.24)	96.9	0.000
BMI Match				
Yes	10	1.40(0.90,1.91)	91.0	0.000
No	5	0.70(0.50,0.90)	39.9	0.150

Abbreviations: NOS = Newcastle-Ottawa Scale; SMD = Standardized Mean Difference; CI = Confidence Interval; BMI = Body Mass Index

### Heterogeneity analysis

**Figure 3:**
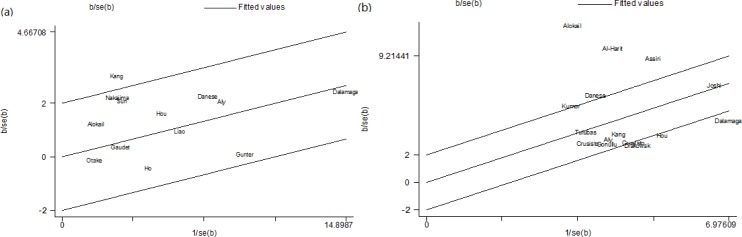
Galbraith plots of the association between circulating resistin levels and obesity-related cancer risk in pooling ORs analysis (a) and pooling SMD analysis of retrospective studies (b)

Sensitivity analysis was conducted to test the robustness of the results of meta-analysis by omitting one study every time. Results showed remaining studies yielded consistent results in pooling both ORs and SMD analysis (Figure S1). Galbraith plot analysis was used to spot the outliners as the potential sources of heterogeneity. For the pooling ORs analysis, one study was identified as the outlier and possible major source of heterogeneity [14] (Figure 3a). Except for the study, the association between resistin levels and cancer risk was still significant (OR = 1.18, 95% CI = 1.09-1.28, *I^2^* = 5.0%, *P* (for heterogeneity analysis) = 0.396) (Figure S2a). For the pooling SMD analysis of retrospective studies, three studies were identified as major contributors to high heterogeneity [9, 12, 22, 23] (Figure 3b). After excluding those studies, high resistin levels were still found in cancer patients (SMD = 0.75, 95% CI = 0.63-0.87, *I^2^* = 39.3%, *P* (for heterogeneity analysis) = 0.087) (Figure S2b). Furthermore, exploratory univariate meta-regression analysis was performed with sample size, cancer type, geographic area, detection assay, study design, study quality, and BMI-match as the covariates. For pooling ORs analysis, geographic area (*P* = 0.019, adjusted R^2^ = 50.02%), detection assay (*P* = 0.041, adjusted R^2^ = 86.15%), and study design (*P* = 0.044, adjusted R^2^ = 13.31%) were found to be significant factors (Table 3). For pooling SMD analysis of retrospective studies, meta-regression analysis revealed geographic area, BMI-match, size, and quality score could account for 66.7% between-study variance (tau2 from 0.547 to 0.182).

### Estimation of publication bias

**Figure 4:**
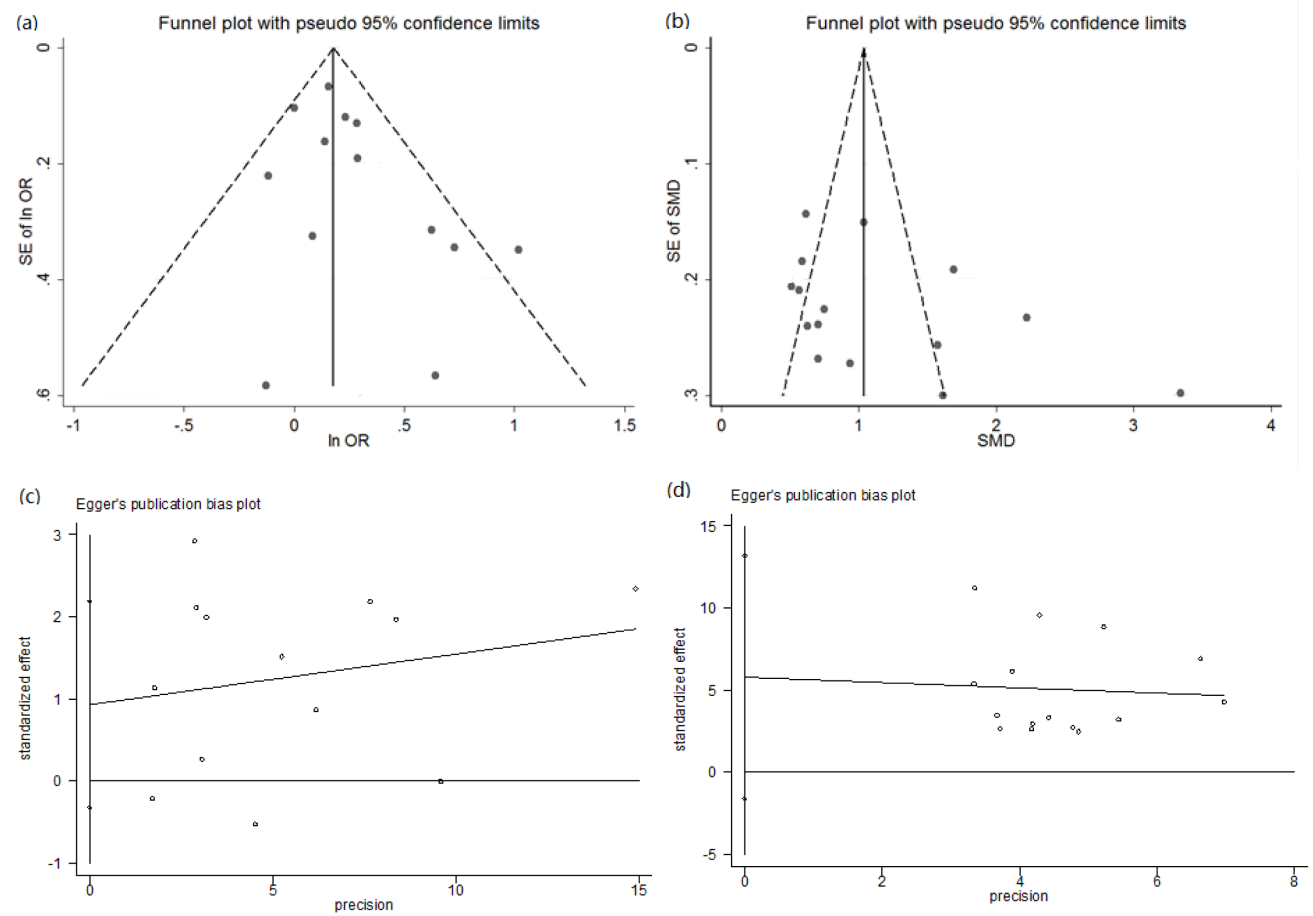
The funnel plots and Egger's bias plot of publication bias in pooling ORs analysis (a and c) and pooling SMD analysis of retrospective studies (b and d)

Publication bias was examined by visual inspection of funnel plots and Egger's regression asymmetry test. For pooling ORs analysis, the shape of funnel plots did not indicate any evidence of publication bias (Figure 4a). Egger's regression test further confirmed this (*P* = 0.180) (Figure 4c). For pooling SMD analysis, the funnel plot had an asymmetrical distribution (Figure S3a). Egger's regression test also showed there was publication bias (*P* = 0.001) (Figure S3b). For pooling SMD analysis of retrospective studies, funnel plots had a slightly asymmetrical distribution (Figure 4b). However, Egger's regression test suggested publication bias was insignificant (*P* = 0.150) (Figure 4d).

## DISCUSSION

Currently, increased attention has been paid to the role of resistin in obesity-related cancers. Whether circulating resistin levels are higher in cancer patients is inconsistent. A meta-analysis was conducted by pooling both ORs and SMD. Higher resistin levels were found to be associated with increased obesity-related cancer risk. Serum resistin levels may be an independent risk of obesity-related cancers, but not a predictor. It may be the first attempt to synthesize the existing studies to evaluate the association of circulating resistin levels with obesity-related cancer risk.

It is widely accepted that increased BMI and insulin resistance are associated with increased obesity-related cancer risk. Resistin was considered as an adipocytokine secreted by adipocytes, monocytes and macrophages, especially in the visceral adipose tissue. Increasing evidence has shown human resistin could stimulate the production of pro-inflammatory cytokines and was an inflammatory biomarker [31, 32]. Chronic inflammation plays an important role in tumorigenesis. It seems plausible that resistin levels may be associated with the incidence of obesity-related cancer. However, the results of clinical trials are not always consistent. Meta-analysis allows a much greater possibility of reaching reasonably strong conclusions. The results of our meta-analysis suggested circulating resistin levels were higher in obesity-related cancer patients and an independent risk factor of obesity-related cancers. For pooling ORs analysis, stratification analysis showed significance only in those studies with colorectal cancer, breast cancer, ELISA detection assay, Asia and Europe, and retrospective studies. There was a lack of strong association in the studies of Human Adipokine Panel detection assay, the USA, and prospective studies. For the pooling SMD analysis, the association was also insignificant only in prospective studies. The prospective studies were mostly conducted in the USA, and detected by Human Adipokine Panel, while most of the retrospective studies were performed in Asia and Europe, and used ELISA to detect serum or plasma resistin levels. For prospective studies, the blood for resistin detection was drawn at the baseline of the follow-up. At that time, all subjects including those becoming cases later were still free of cancer. For retrospective studies, blood was collected when patients were diagnosed with cancer. This may contribute greatly to the differences of results between retrospective studies and prospective studies. It indicates circulating resistin levels may not be a predictor of obesity-related cancers at least in the USA. Prospective studies need to be conducted in Asia and Europe, and retrospective studies need to be conducted in the USA.

The heterogeneity of between-study is common in meta-analysis. Meta-analysis showed significant between-study heterogeneity, especially in pooling SMD analysis. Sensitivity analysis, subgroup analysis, Galbraith plots, and meta-regression analysis were used to explore the potential causes of between-study heterogeneity and to reduce the heterogeneity. Sensitivity analysis didn't find any single study affected the estimated significance of pooled ORs or SMD. Galbraith plots indicated that 1 outlier study contributed to heterogeneity in pooling ORs analysis, while 4 outlier studies contributed to heterogeneity in pooling SMD analysis of retrospective studies. The results of the outlier studies greatly deviated from the pooling results. After omitting these studies, heterogeneity was insignificant. The pooling results didn't change significantly because of excluding the outlier studies. Meta-regression analysis found factors such as geographic area, detection assay, or study design almost completely accounted for some between-study variance in pooling ORs analysis, while geographic area, BMI-match, size, and quality score contributed significantly to heterogeneity of between-study in pooling SMD analysis of retrospective studies.

However, some limitations in the meta-analysis should be demonstrated, and the results should be prudently explained. First, our meta-analysis was limited to articles published in English. Slight publication bias may exist, especially for pooling SMD analysis. Some eligible articles may have been missed. Second, most studies included in our meta-analysis were case-control studies. It's widely known that case-control studies have inherent limits, such as selection bias, admission rate bias, detection signal bias. Third, the confounding factors in the studies for ORs analysis were inadequately considered due to various adjustments made in studies and some potential confounders were not considered in the majority of studies, such as diseases other than cancer, inflammatory conditions, drugs, and hormonal factors. Additionally, it should be noted that remarkable heterogeneity existed in pooling SMD analysis, and may have reduced the reliability of the meta-analysis.

In conclusion, this meta-analysis suggests circulating resistin levels may be higher in obesity-related cancer patients than in normal controls, and an independent biomarker of obesity-related cancer risk. But it may not be a predictor of obesity-related cancer risk. Given the limited number of studies included as well as the significant heterogeneity, more randomized and large-scale clinical trials, carefully controlled for potential confounding factors, are needed to confirm this association between resistin levels and obesity-related cancer risk in the future.

## MATERIALS AND METHODS

### Search strategy

A comprehensive literature screening was conducted for publications up to February 20th, 2016 from the following databases: (1) Pubmed (http://www.ncbi.nlm.nih.gov/pubmed/); (2) Embase (https://www.embase.com/); (3) Cochrane (http://www.cochranelibrary.com/). Search terms: “resistin, RETN”, “cancer, tumor, neoplasm, carcinoma” and “serum, plasma, circulating, blood” were used in combination to retrieve the relevant literatures. Only papers written in English language were considered in this study. In addition, reference lists of articles were scrutinized to identify additional articles. This study was planned and conducted in accordance with standards of quality for reporting meta-analysis [33].

### Eligibility criteria

Only studies meeting the following criteria were included: (1) the study must be an original epidemiological study; (2) the exposure of interest must be the serum or plasma resistin detected in blood samples; (3) the outcome of interest must be concerned with obesity-related cancers, including prostate, breast, colorectal, thyroid, renal, endometrial, pancreatic and esophageal cancers; (4) the study must report odds ratio (OR) or relative risk (RR), corresponding 95% confidence intervals (CI), mean and standard deviation (SD), or data to calculate these. Studies that did not refer to cancer, serum or plasma resistin, healthy controls, and that were conducted on animals, cells, or tissues were excluded. Two investigators (Wei Zheng and Wei-Jing Gong) reviewed all studies independently to identify and determine whether an individual study was eligible for inclusion in this meta-analysis. Any disagreement between the studies was resolved by consensus with a third reviewer (Zhao-Qian Liu).

### Data extraction

Data was extracted and assessed by two independent researchers (Li-Ming Tan and Wei-Jing Gong) using the Newcastle-Ottawa Scale (NOS). Disagreements were resolved by consensus. Data extracted from eligible studies included first author's last name, year of publication, country of origin, study design, BMI, age, cancer type, sample size, resistin detection assay, confounders adjusted in multivariate analysis, RR or OR with corresponding 95%CI for the risk of cancer incidence, mean and SD, or data to calculate them [34, 35].

### Statistical analysis

Heterogeneity of effect size among studies was assessed by the Cochrane's *Q*-statistic test and *I^2^* test. If *P* < 0.05 and *I^2^*> 50%, a random effect model was used, otherwise, a fixed effect model was used [36, 37]. Sensitivity analysis was performed to assess the influence of a single study on the summary results. When heterogeneity was present, subgroup analysis, Galbraith plot and meta-regression analysis were used to detect the potential sources of heterogeneity [38, 39]. Funnel plots and Egger's test were carried out to estimate publication bias [40, 41]. Statistical analyses were performed using STATA version 12 (Stata Corp, College Station, TX, USA), and tests were two-sided with the criterion of statistical significance at *P* < 0.05.

## SUPPLEMENTARY MATERIAL FIGURES


