# Supplementary material for: Circulating resistin levels and obesity-related cancer risk: A meta-analysis
Source: Oncotarget. 2016 Aug 4;7(36):57694–704. doi: 10.18632/oncotarget.11034 (PMC5295382; doi:10.18632/oncotarget.11034)
Supplement: Supplementary file 1 [file oncotarget-07-57694-s001.pdf]

Circulating resistin levels and obesity-related cancer risk: a meta-analysis

Supplementary Material

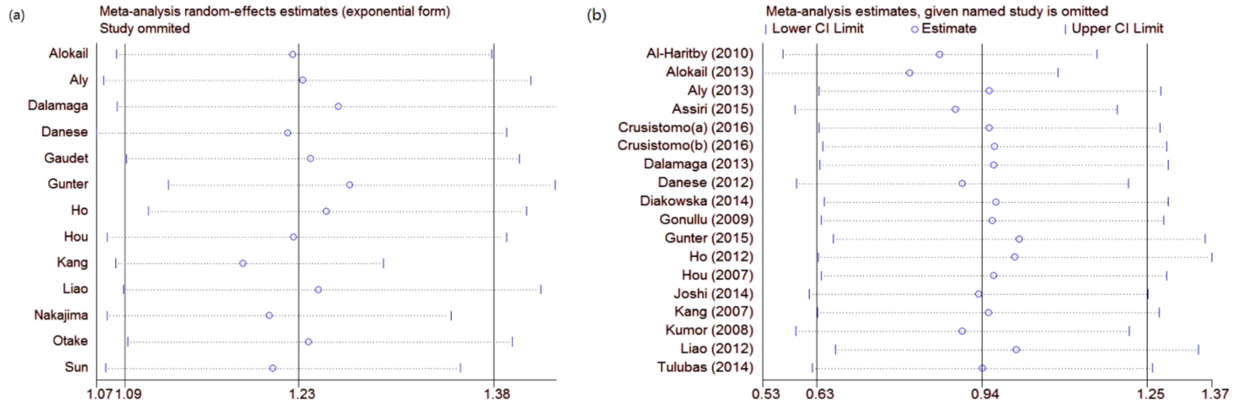

Figure S1: The effect of circulating resistin levels on obesity-related cancer risk in pooling ORs (a) analysis and pooling SMD (b) analysis after omitting outlier studies" by "The effect of circulating resistin levels on obesity-related cancer risk in pooling ORs analysis (a) and pooling SMD analysis of retrospective studies (b) after omitting outlier studies estimate

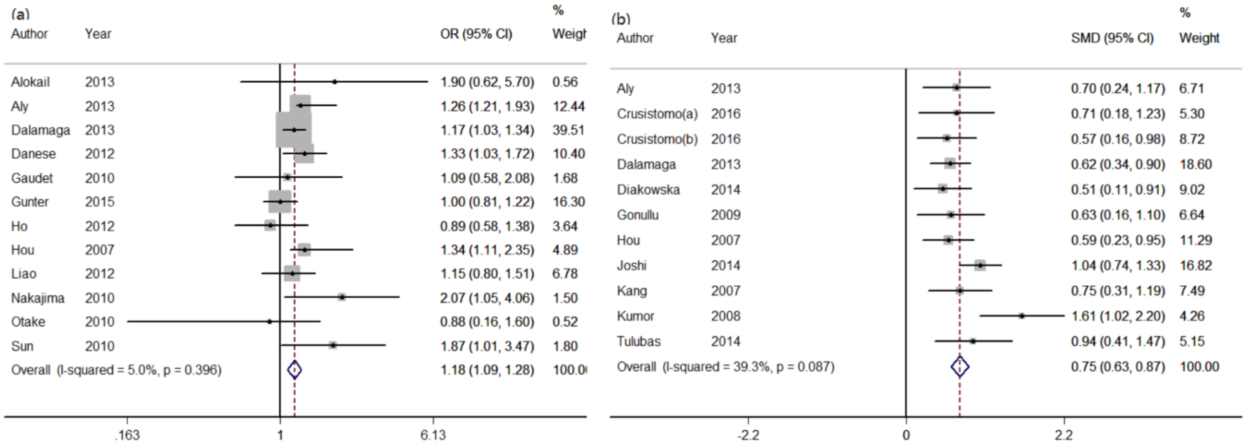

Figure S2: The effect of circulating resistin levels on obesity-related cancer risk in pooling ORs analysis (a) and pooling SMD analysis of retrospective studies (b) after omitting outlier studies

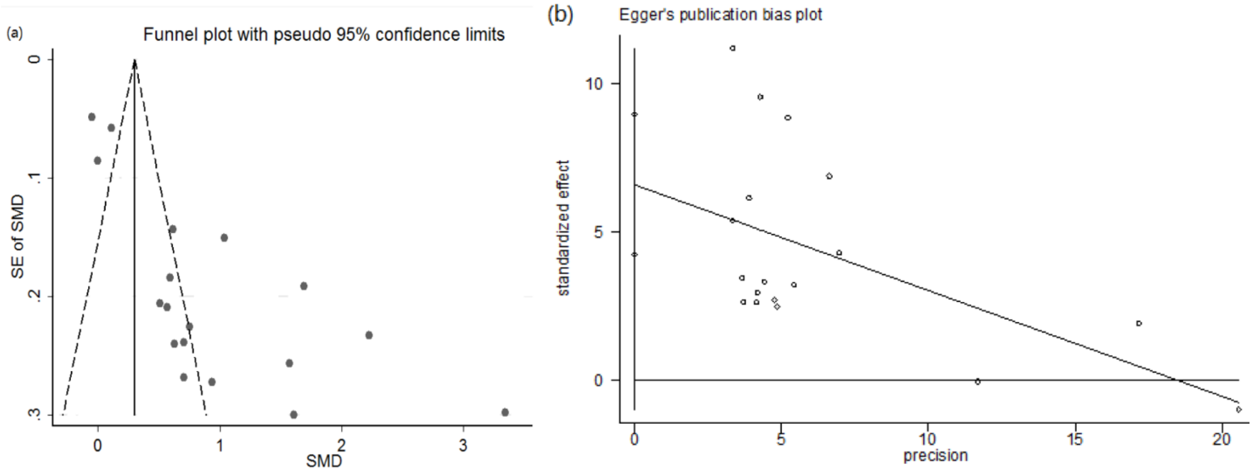

Figure S3: The funnel plots (a) and Egger's bias plots (b) of publication bias in pooling SMD analysis
